# A functional variant at *miRNA*-122 binding site in *IL-1α* 3′ UTR predicts risk of recurrence in patients with oropharyngeal cancer

**DOI:** 10.18632/oncotarget.8908

**Published:** 2016-04-21

**Authors:** Chengyuan Wang, Erich M. Sturgis, Xingming Chen, Qingyi Wei, Guojun Li

**Affiliations:** ^1^ Department of Head and Neck Surgery, The University of Texas MD Anderson Cancer Center, Houston, TX 77030, USA; ^2^ Department of Otolaryngology-Head and Neck Surgery, China-Japan Friendship Hospital, Beijing 100029, China; ^3^ Department of Epidemiology, The University of Texas MD Anderson Cancer Center, Houston, TX 77030, USA; ^4^ Department of Otolaryngology-Head and Neck Surgery, Peking Union Medical College Hospital, Peking Union Medical College and Chinese Academy of Medical Sciences, Beijing 100730, China; ^5^ Duke Cancer Institute, Duke University Medical Center, Durham, NC 27710, USA

**Keywords:** IL1a, recurrence, oropharyngeal cancer, HPV, biomarker

## Abstract

*IL-1α*, an important regulator of immune and inflammation responses, has been implicated in cancer development and prognosis. An insertion (Ins)/deletion (Del) polymorphism (*IL-1α* rs3783553) in the 3′ UTR of *IL-1α* may disrupt a binding site for miRNA-122 and may affect its transcription level. Thus, this polymorphism may cause interindividual variation in immune and inflammation responses and thus may lead to different susceptibility to treatment response and prognosis of such patients. We evaluated the association of *IL-1α* rs3783553 polymorphism with risk of recurrence of squamous cell carcinoma of the oropharynx (SCCOP) in a cohort of 1008 patients. Log-rank test and univariate and multivariable Cox models were used to evaluate associations. Compared with patients with Del/Del homozygous genotype, the patients with Ins/Del+Ins/Ins variant genotypes had worse disease-free survival (log-rank *P* < 0.0001) and increased risk of SCCOP recurrence (HR, 2.4, 95% CI, 1.7-3.3) after multivariable adjustment. Furthermore, among patients with HPV16-positive tumors, the patients with Ins/Del+Ins/Ins variant genotypes of the *IL-1α* polymorphism had worse disease-free survival (log-rank *P* < 0.0001) and much higher recurrence risk than those with Del/Del homozygous genotype of this polymorphism (HR, 16.3, 95% CI, 5.0-52.7). Our findings suggest that *IL-1α* rs3783553 polymorphism may modulate the risk of SCCOP recurrence in patients, particularly for patients with HPV16-positive tumors. However, larger studies are needed to validate these results.

## INTRODUCTION

Despite declining smoking rates in the United States, the incidence of squamous cell carcinoma of the oropharynx (SCCOP), a subsite of squamous cell carcinoma of the head and neck (SCCHN), is increasing [1]. The growing incidence of SCCOP may be mainly attributed to the increasing prevalence of infection with high-risk types of human papillomavirus (HPV), particularly HPV16 [2-4]. While surgery, radiotherapy, and chemotherapy have been used for treatment of SCCOP, the long-term survival of patients with SCCOP has improved only moderately. The one of the main reasons for low survival rate is due to the high rate of recurrence as patients with disease recurrence after multimodal treatment have a less than 5% rate of five-year survival [5, 6]. The recurrence rates of SCCOP may differ among patients with similar clinical characteristics and similar therapeutic approaches, indicating that it is likely that other factors, such as genetic factors, may also contribute to risk of recurrence of SCCOP. Therefore, accurate prediction of recurrence risk in individual patients with SCCOP would help clinicians to ensure appropriate individualized treatment, which might lead to improved survival and better quality of life.

The host immune and chronic inflammation responses play important roles in HPV infection and treatment response to radiotherapy. Persistent HPV infection may affect disease development and clinical outcome, suggesting the important roles of the host immune and inflammation responses in treatment of SCCOP, particularly in HPV-related tumors. Interleukin (IL)-1α plays an important role in the regulation of immune response and the defense against viral infections [7]. Several studies have suggested that IL-1 either promotes or blocks the processes of tumorigenesis, tumor proliferation, angiogenesis, invasion, and metastasis [8-14]. Woodworth et al found that IL-1α inhibited the proliferation of normal epithelial cells cultured from human cervix tissues [15], while IL-1α significantly stimulated the proliferation of cervical cell lines immortalized by transfection with HPV16 or HPV18 DNA. Other studies found that gene expression for IL-1α was decreased in HPV16- or HPV18-associated cervical squamous cell carcinoma samples and HPV-infected cells [16, 17]. Given the crucial and conflicting roles of IL-1α in immune and inflammation regulation [7, 15-19], its genetic variants may affect the host immune an inflammatory responses and, subsequently the sensitivity to radiotherapy and prognosis.

MiRNA have important roles in a broad range of biological processes, such as embryonic development, cellular differentiation, proliferation, apoptosis, and cancer development [20-22]. To regulate mRNA level and protein expressions, miRNAs bind to targeted mRNA in the 3′ UTR. Thus, polymorphisms in the 3′ UTR targeted by miRNAs can either abolish existing binding sites or create illegitimate binding sites, which results in the regulation of target genes that can affect expression of target genes [23, 24]. An insertion/deletion polymorphism (rs3783553, an insertion or deletion of TTCA bases) at the miRNA-122 binding site, which is located in the *IL-1α* 3′ UTR, was shown to be associated with gastric, hepatocellular, nasopharyngeal, and thyroid carcinomas [12, 14, 20, 21, 24]. Our previous study has found that *IL-1α* 3′ UTR rs3783553 polymorphism may be functional and influence susceptibility to HPV16-associated OSCC, particularly for SCCOP. However, no larger studies have examined the associations between the *IL-1α* rs3783553 polymorphism and risk of recurrence of patients with SCCOP. Therefore, we hypothesized that the *IL-1α* rs3783553 polymorphism at miRNA-122 binding site in *IL-1α* 3′ UTR is associated with risk of recurrence of SCCOP, particularly HPV16-associated SCCOP. In the present study we assessed the association in a cohort of 1008 incident patients with SCCOP.

## RESULTS

We had a total of 1,236 SCCOP patients from May 1995 to October 2013, and there were 230 patients were excluded from the final analysis. We excluded these patients from final analysis because they were not incident patients; had no follow-up and treatment status; and no blood samples available for the study. We also compared the distribution of the characteristics in Table 1 between the 1008 study patients and the 230 excluded patients, the differences were not statistically significant (all P > 0.05) except for sex with a borderline significance (*P* = 0.057). Thus, the present study included a final cohort of 1008 patient who were followed up, with an overall median follow-up time of 44.7 months (range, 1.7 to 170.9 months); 181 patients experienced disease recurrence. The median follow-up durations for patients without and with recurrence were 50.9 and 11.6 months, respectively. Of the 181 patients with recurrence, 70 (38.7%) had distant recurrence, 49 (27.1%) had local recurrence, 20 (11.0%) had regional recurrence, and 42 (23.2%) had recurrence of more than one type. The mean ages at diagnosis for the overall cohort, patients who developed recurrence, and patients without recurrence were 55.8, 58.6, and 55.2 years, respectively.

**Table 1:** Characteristics of patients with SCCOP (N = 1008)

Variable	No. (%) of patients	No. of patients with recurrence	5-year recurrence rate (%)	*P *^a^ value
No. of patients	1008 (100)	181	0.20	
Age				
≤ 57 years	621 (61.6)	85	0.15	< 0.0001
> 57 years	387 (38.4)	96	0.27	
Sex				
Male	872 (86.5)	161	0.20	0.3110
Female	136 (13.5)	20	0.19	
Ethnicity				
Non-Hispanic white	913 (90.6)	146	0.17	< 0.0001
Other	95 (9.4)	35	0.41	
Smoking				
Never	388 (38.5)	51	0.14	0.0004
Ever	620 (61.5)	130	0.23	
Alcohol use				
Never	247 (24.5)	26	0.10	0.0005
Ever	761 (75.5)	155	0.23	
Comorbidity				
None or mild	913 (90.6)	157	0.19	0.0370
Moderate to severe	95 (9.4)	24	0.27	
Index cancer stage				
1 or 2	72 (7.1)	11	0.19	0.5280
3 or 4	936 (92.9)	170	0.20	
Treatment				
X/XC/XS/S	947 (93.9)	166	0.19	0.0030
SXC	61 (6.1)	15	0.32	

a *P*: Log-rank test for DFS between the two groups.

X, radiotherapy; C, chemotherapy; and S, surgery.

Table 1 presents patients' demographic, risk, and clinical factors and the corresponding 5-year actuarial recurrence rates. Patients in the overall group were predominantly male (86.5%) and non-Hispanic white (90.6%). The univariate Kaplan-Meier analyses showed that age, ethnicity, smoking, alcohol use, comorbidity, and treatment were significantly associated with DFS (all *P* < 0.05), whereas significant associations were not found for sex and index cancer stage (all *P* > 0.05).

**Figure 1:**
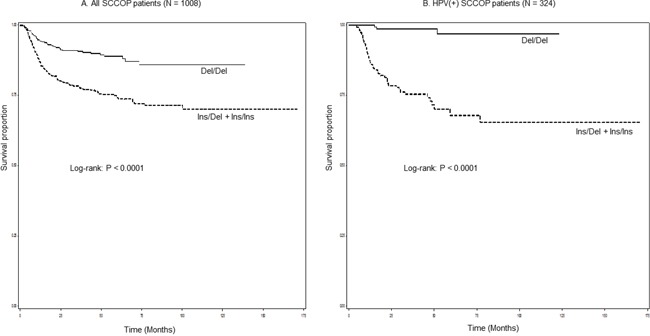
Kaplan-Meier estimates for the cumulative recurrence rates of patients according to *IL-1α* rs3783553 genotypes (A. all SCCOP patients and B. Tumor HPV16-positive SCCOP patients)

Table 2 shows the genotype distributions of the *IL-1α 3′ UTR rs3783553* polymorphism, 5-year actuarial recurrence rates, and results of univariate and multivariable analyses of the associations of the polymorphism with recurrence risk. The patients with Ins/Ins or Ins/Del genotype had a significantly worse DFS than those with in patients with Del/Del genotype (log-rank *P* < 0.0001) (Figure 1A). Multivariable Cox proportional hazards regression analysis was performed to evaluate the association between the *IL-1α 3′* UTR rs3783553 polymorphism and recurrence risk in patients with SCCOP. The adjusted confounders included age, sex, ethnicity, smoking status, alcohol status, comorbidity, stage, and treatment. As shown in Table 2, compared with those with Del/Del genotype, the patients with Ins/Ins or Ins/Del genotype had a significantly increased risk of disease recurrence (HR, 2.4, 95% CI, 1.7-3.3).

**Table 2:** Association between *IL-1α* rs3783553 genotypes and SCCOP recurrence in patients with SCCOP (N = 1008)

Genotype	No. of recurrences/No. of patients	5-year recurrence rate	Log-rank *P* value	aHR^*^, 95% CI
*IL-1α* rs3783553			< 0.0001	
Del/Del^†^	45/444	0.10		1.0
Ins/Del + Ins/Ins	136/564	0.25		2.4 (1.7-3.3)

HR, hazard ratio

* Adjusted for age, sex, ethnicity, smoking status, alcohol use status, stage, comorbidity, and treatment.

† Reference group

Because HPV is an important prognostic factor for SCCOP and because IL-1α is one of important regulators in inflammation response, we further explored the associations between *IL-1α 3′* UTR rs3783553 polymorphism and risk of recurrence of SCCOP patients with HPV16-positive tumors. Since we found that the patients with common homozygous Del/Del genotype of the *IL-1α 3′ UTR rs3783553* polymorphism were approximately three times as likely as patients with the corresponding Ins/Ins+Ins/Del variant genotypes to have HPV16-positive tumors (OR, 3.2, 95% CI, 1.9-5.7) (data not shown), we then performed univariate and multivariable analyses to determine the effect of *IL-1α 3′* UTR rs3783553 polymorphism on risk of recurrence among 324 SCCOP patients with HPV16-positive tumors. As shown in Figure 1B, a significantly worse DFS was found in patients with *IL-1α 3′ UTR rs3783553* Ins/Ins+Ins/Del genotypes than in those with corresponding Del/Del genotype (log-rank P < 0.0001). Furthermore, multivariable Cox proportional hazards regression analysis (Table 3) showed that the patients with *IL-1α 3′* UTR rs3783553 Ins/Ins+Ins/Del genotypes had an approximately 16.5-fold significantly increased risk for recurrence compared with those with the corresponding Del/Del genotype (HR, 16.3, 95% CI, 5.0-52.7). Additionally, we did not assess the similar associations between the *IL-1α 3′* UTR rs3783553 polymorphism and recurrence risk among the patients with HPV16-negative tumors, since we did not have enough sample size or number of outcome events in these patients for meaningful statistical analysis.

**Table 3:** Association between *IL-1α* rs3783553 genotypes and HPV-positive SCCOP recurrence in patients with SCCOP (N = 324)

Genotype	No. of recurrences/No. of patients	5-year recurrence rate	Log-rank *P* value	aHR^*^, 95% CI
*IL-1α* rs3783553			< 0.0001	
Del/Del^†^	3/171	0.03		1.0
Ins/Del + Ins/Ins	42/153	0.30		16.3 (5.0-52.7)

HR, hazard ratio

* Adjusted for age, sex, ethnicity, smoking status, alcohol use status, stage, comorbidity, and treatment.

† Reference group

## DISCUSSION

In this cohort study of 1008 patients with incident SCCOP, we found that the *IL-1α* 3′ UTR rs3783553 Ins/Ins+Ins/Del genotypes were significantly associated with an increased risk of recurrence; furthermore, we found that these Ins/Ins+Ins/Del genotypes were significantly associated with the increased risk of recurrence among SCCOP patients with HPV16-positive tumors. These results suggest that the *IL-1α 3′ UTR* rs3783553 polymorphism may predict risk of recurrence among SCCOP patients, particularly for those with HPV16-posiitve tumors.

To date, this is the first epidemiological study to assess the association between the rs3783553 polymorphism and risk of recurrence of SCCOP. Although we do not know how this *IL-1α* 3′ UTR variant influences the recurrence risk of tumor HPV16-positive patients, it is biologically plausible that this variant may be either functional or in linkage disequilibrium with other functional variants of *IL-1α*, thereby altering the function of *IL-1α*, or with alleles at other nearby susceptibility loci. Such functional variants could increase or reduce *IL-1α* expression levels and thus affect the regulation of the immune and inflammation as well as inflammatory or apoptotic responses. For example, *IL-1α* 3′ UTR rs3783553 Del/Del genotype might alter regulation in these pathways which might enable many HPV-infected cells to escape or counterattack against the immune system and might not enhance apoptotic response, leading to more likely to be HPV-positive tumors and subsequently better response to radiotherapy [7]. Therefore, this *IL-1α* polymorphism may serve as a prognostic biomarker for clinical outcome of SCCOP.

Growing evidences have suggested that the polymorphisms in the miRNA target site may influence the strength of miRNA binding, regulation of target genes and affecting the individual's cancer risk [25, 26]. The rs3783553 lies within a predicted binding site (seed region) for human miR-122, which is a liver specific miRNA comprising up to 70% of all hepatic miRNA which mostly regulates lipid homeostasis [27-29]. MiR-122 was found to be downregulated in hepatocellular carcinoma with a function of tumor suppressor [30]. Furthermore, Gao et al. reported the rs3783553 polymorphism affects the transcription of IL-1α by altering the binding strength of miRNA-122 [21]. Subsequently, this polymorphism has been identified to be associated with decreased risks for developing hepatocellular carcinoma, nasopharyngeal carcinoma, gastric cancer, papillary thyroid carcinoma, and cervical cancer [12, 14, 20, 21, 31]. More recently, a case-control study has reported the associations between this polymorphism and risk of SCCOP, particularly the risk of HPV16-associated SCCOP, which suggests a joint effect of the *IL-1α* polymorphism and HPV16 infection on risk of SCCOP [32].

Since this polymorphism is within the functional region of the gene's 3′UTR of *IL-1α*, we speculated that this *IL-1α* genetic variant may have potentially functional effect on expression levels of *IL-1α* by altering the efficiency of translational initiation, leading to inter-individual differences in susceptibility to radiotherapy. Indeed, we previously reported that the Del/Del genotype of this polymorphism is significantly correlated with increased expression of *IL-1α* in serum. While the functional relevance of this polymorphism has not yet been elucidated, our results might partially suggest a functional correlation between this polymorphism and expression of *IL-1α*, which may provide preliminary evidence of biological plausibility for the observed association in the current study.

Since IL-1α plays an important role in immune and inflammatory responses, we thus further explored the roles of *IL-1α* 3′ UTR rs3783553 in recurrence of SCCOP patients stratified by tumor HPV16 status. We found that the modifying effect of the *IL-1α* 3′ UTR rs3783553 polymorphism on SCCOP recurrence was more pronounced in SCCOP patients with HPV16-positive tumors. These results suggest that *IL-1α* 3′ UTR rs3783553 polymorphism may modulate risk of recurrence in patients with HPV16-positive tumors. The mechanism behind such results among patients with SCOOP is not fully understood. We expect that the *IL-1α* 3′ UTR rs3783553 variant genotypes may result in decreased expression of *IL-1α* (25), which may confer protective effects against HPV infection among patients with SCOOP. This *IL-1α* 3′ UTR rs3783553 polymorphism has been reported to affect the level of IL-1α [21]. The decreased levels of IL-1α may decrease immune and inflammation responses to HPV16 infection, leading to decreased HPV clearance capacity and increased risk of persistent HPV16 infection. Furthermore, decreased levels of *IL-1α* can fail to arrest growth of HPV-infected cells and deduce apoptosis of HPV-infected cells. Finally, Patients with HPV16-positive tumors are more likely to have intact p53 rather than mutated p53. These patients might have apoptosis in response to genotoxic injuries, such as radiotherapy. Therefore, the *IL-1α* 3′ UTR rs3783553 variant genotypes may reduce the inflammation and p53-induced apoptotic responses among patients with HPV16-positive tumors, leading to worse response to definitive radiotherapy and subsequently the increased likelihood of disease recurrence. However, these hypotheses need to be verified in future studies.

Although this study reveals some significant associations between *IL-1α* 3′ UTR rs3783553 polymorphism and recurrence risk of SCCOP, there are several limitations including lack of details on radiotherapy (e.g., dosage, treatment cycles, duration, etc), the limited number of outcome events in HPV16-positive, multiple ethnicities inclusion, and the relatively short follow-up time, as well as the hospital-based nature of the study. In conclusion, our findings suggest that the *IL-1α* 3′ UTR rs3783553 polymorphism might modulate the risk of recurrence of SCCOP, especially in patients with HPV16-positive tumors. Therefore, to confirm the validity and utility of these polymorphisms as clinical prognostic biomarkers, future prospective well-designed larger studies are needed to validate our results and further explore the molecular mechanisms underlying the observed associations.

## MATERIALS AND METHODS

### Study subjects

The cohort of patients with incident SCCOP for this study were consecutively enrolled from May 1995 to October 2013 as described previously [33]. Briefly, all patients had newly diagnosed, histopathologically confirmed, previously untreated SCCOP and were recruited without discrimination regarding age, sex, ethnicity, or clinical stage except that patients with distant metastases on presentation were excluded. All subjects signed an informed consent form that was approved by the Institutional Review Board of The University of Texas MD Anderson Cancer Center. Approximately 95% of contacted patients consented to enrollment in the study. For the analysis described in this paper, some participants were excluded if they 1) had known distant metastases; 2) had any prior cancer except nonmelanoma skin cancer; 3) had a primary sinonasal tumor, a salivary gland tumor, cervical metastases of unknown origin, or a tumor outside the upper aerodigestive tract; 4) had no blood samples available for genotyping (this was the case for some patients who were recruited early in the parent study); 5) had treatment performed outside of our institution; or 6) underwent only palliative treatment.

Patients were monitored through their treatment and posttreatment course with scheduled regular clinical and radiographic examinations. Patients were considered disease free if absence of disease was documented at the date of the last visit with the head and neck surgeon, head and neck radiation oncologist, or head and neck medical oncologist. There were no universal standards for imaging. Typically, patients had either routine serial imaging or follow-up imaging on the basis of symptoms or findings on physical examination. Patients were regarded as having disease recurrence if they experienced appearance of a new lesion of the same histology verified by biopsy (incisional, excisional, or needle biopsy) and reappearance of any lesion that had disappeared.

Clinical data, such as stage at presentation of the index tumor, treatment, comorbidity, and recurrence, were obtained from review of the medical records. The sixth edition of the American Joint Committee on Cancer TNM staging system was used to determine disease stage at the time of presentation for all study patients. Definitive radiotherapy was defined if the patients received radiation treatment only or radiation treatment in combination with any other therapeutic modalities. Medical comorbidities were classified according to a modification of the Kaplan-Feinstein comorbidity index (Adult Comorbidity Evaluation 27), which reflects the presence of related comorbidities as none to mild, moderate, or severe [34]. Data on alcohol use and smoking status were collected at the initial presentation. “Ever drinkers” were defined as patients who had drunk at least one alcoholic beverage per week for at least 1 year during their lifetime, and patients who had never had such a pattern of drinking were considered “never drinkers.” Patients who had smoked at least 100 cigarettes in their lifetime were defined as “ever smokers,” and patients who had smoked fewer than 100 cigarettes in their lifetime were categorized as “never smokers.”

### IL-1α 3′ UTR rs3783553 genotyping

For this study, we extracted genomic DNA from a leukocyte cell pellet using the QIAamp DNA Blood Mini Kit (QIAGEN Inc., Valencia, CA) in accordance with the manufacturer's instructions. Genotyping using a polymerase chain reaction (PCR) assay was performed as previously described [21]. DNA fragments containing the polymorphism were amplified with the forward primer 5′-ATTGGTCCGATCTTTGACTC-3′ and reverse primer 5′-TGATAA CAGTGGTCTCATGG-3′. The PCR products were analyzed by 6% non-denaturing polyacrylamide gel electrophoresis and visualized by silver staining. The genotypes were determined by the numbers and the lengths of the bands in the gels. Repeat analysis was performed on a randomly selected subset of 10% of the samples, and the results were in 100% concordance with the initial analysis.

### Tumor HPV16 detection

Paraffin-embedded tissue biopsies or specimens from study patients with tissues available were used to extract DNA for tumor HPV16 detection using the specific PCR and in situ hybridization methods as described previously [35]. For quality control, a subset of samples were re-assayed for tumor HPV16 status. The results of the re-run samples were 100% concordant with the original results.

### Statistical analysis

We first used Student's *t* test to compare the mean age and follow-up time for patients with and without recurrence. The primary endpoint of the study was recurrence. Time to event was calculated from the date of diagnosis of the index SCCOP to the date of clinically detectable recurrence (local, regional, or distant). Patients who were not known to have had an event at the date of last contact and patients who were lost to follow-up or died of other/unknown cause were censored. The associations between individual epidemiologic risk factors, clinical characteristics (including stage, comorbidity, and treatment variables), and time to recurrence were initially assessed using univariate Cox proportional hazards regression models.

The associations between individual epidemiological factors, clinical characteristics, and treatment variables, and time to recurrence, were initially assessed using univariate Cox proportional hazards regression models. The data were consistent with the assumptions of the Cox proportional hazards regression model from the examination of Kaplan-Meier survival curves and log-minus-log survival plots. The associations between variables and disease-free survival (DFS) were evaluated using the log-rank test. We assessed the associations between individual epidemiologic risk factors, clinical characteristics (including stage, comorbidity, and treatment variables), and time to recurrence using both univariate and multivariable Cox proportional hazards regression models. Associations between *IL-1α 3′ UTR rs3783553* polymorphism and risk of recurrence were quantified by calculating hazard ratios (HRs) and their 95% CIs. The Cox model included adjustment for potential confounders, including age, sex, ethnicity, smoking status, alcohol use status, tumor stage, comorbidity, and treatment. For all analyses, statistical significance was set at *P* < 0.05, and all tests were two-sided. SAS software (version 9.2.3; SAS Institute) was used to perform all statistical analyses.

## Abbreviations

IL-1α: Interleukin-1α
UTR: Untranslated regions
SCCHN: squamous cell carcinoma of the head and neck
SCCOP: squamous cell carcinoma of the oropharynx
HR: Hazard ratio
CI: confidence interval
PCR: polymerase chain reaction
SNP: single nucleotide polymorphism

## References

[1] Chaturvedi AK, Engels EA, Anderson WF, Gillison ML (2008). Incidence trends for human papillomavirus-related and -unrelated oral squamous cell carcinomas in the United States. J Clin Oncol.

[2] Marur S, D'Souza G, Westra WH, Forastiere AA (2010). HPV-associated head and neck cancer: a virus-related cancer epidemic. Lancet Oncol.

[3] Gillison ML, Koch WM, Capone RB, Spafford M, Westra WH, Wu L, Zahurak ML, Daniel RW, Viglione M, Symer DE, Shah KV, Sidransky D (2000). Evidence for a causal association between human papillomavirus and a subset of head and neck cancers. J Natl Cancer Inst.

[4] Ang KK, Harris J, Wheeler R, Weber R, Rosenthal DI, Nguyen-Tân PF, Westra WH, Chung CH, Jordan RC, Lu C, Kim H, Axelrod R (2010). Human papillomavirus and survival of patients with oropharyngeal cancer. N Engl J Med.

[5] Vokes EE, Weichselbaum RR, Lippman SM, Hong WK (1993). Head and neck cancer. N Engl J Med.

[6] Amar A, Chedid HM, Rapoport A, Dedivitis RA, Cernea CR, Brandão LG, Curioni OA (2012). Update of assessment of survival in head and neck cancer after regional recurrence. J Oncol.

[7] Iglesias M, Yen K, Gaiotti D, Hildesheim A, Stoler MH, Woodworth CD (1998). Human papillomavirus type 16 E7 protein sensitizes cervical keratinocytes to apoptosis and release of interleukin-1alpha. Oncogene.

[8] Lee KM, Park SK, Hamajima N, Tajima K, Choi JY, Noh DY (2006). Genetic polymorphisms of interleukin-1 beta (IL-1B) and IL-1 receptor antagonist (IL-1RN) and breast cancer risk in Korean women. Breast Cancer Res Treat.

[9] Mustea A, Sehouli J, Konsgen D, Stengel D, Sofroni D, Lichtenegger W (2003). Interleukin 1 receptor antagonist (IL-1RA) polymorphism in women with cervical cancer. Anticancer Res.

[10] Sehouli J, Mustea A, Koensgen D, Lichtenegger W (2003). Interleukin-1 receptor antagonist gene polymorphism in epithelial ovarian cancer. Cancer Epidemiol Biomarkers Prev.

[11] White KL, Schildkraut JM, Palmieri RT, Iversen ES, Berchuck A, Vierkant RA (2012). Ovarian cancer risk associated with inherited inflammation-related variants. Cancer Res.

[12] Zeng XF, Li J, Li SB (2014). A functional polymorphism in IL-1A gene is associated with a reduced risk of gastric cancer. Tumour Biol.

[13] Lu D, Chen L, Shi X, Zhang X, Ling X, Chen X (2013). A functional polymorphism in interleukin-1alpha (IL1A) gene is associated with risk of alopecia areata in Chinese populations. Gene.

[14] Yang ZH, Dai Q, Zhong L, Zhang X, Guo QX, Li SN (2011). Association of IL-1 polymorphisms and IL-1 serum levels with susceptibility to nasopharyngeal carcinoma. Mol Carcinog.

[15] Woodworth CD, McMullin E, Iglesias M, Plowman GD (1995). Interleukin 1 alpha and tumor necrosis factor alpha stimulate autocrine amphiregulin expression and proliferation of human papillomavirus-immortalized and carcinoma-derived cervical epithelial cells. Proc Natl Acad Sci U S A.

[16] Hu Y, Liu Y, Liu CB, Ling ZQ (2011). Identification of high-risk human papillomavirus (hrHPV)-associated genes in early stage cervical squamous cell carcinomas. J Int Med Res.

[17] Manavi M, Hudelist G, Fink-Retter A, Gschwandtler-Kaulich D, Pischinger K, Czerwenka K (2007). Gene profiling in Pap-cell smears of high-risk human papillomavirus-positive squamous cervical carcinoma. Gynecol Oncol.

[18] Merrick DT, Winberg G, McDougall JK (1996). Re-expression of interleukin 1 in human papillomavirus 18 immortalized keratinocytes inhibits their tumorigenicity in nude mice. Cell Growth Differ.

[19] Hurgin V, Novick D, Werman A, Dinarello CA, Rubinstein M (2007). Antiviral and immunoregulatory activities of IFN-gamma depend on constitutively expressed IL-1alpha. Proc Natl Acad Sci U S A.

[20] Gao L, Zhu X, Li Z, Li L, Wang T, Hu H (2014). Association between a functional insertion/deletion polymorphism in IL1A gene and risk of papillary thyroid carcinoma. Tumour Biol.

[21] Gao Y, He Y, Ding J, Wu K, Hu B, Liu Y, Wu Y, Guo B, Shen Y, Landi D, Landi S, Zhou Y, Liu H (2009). An insertion/deletion polymorphism at miRNA-122-binding site in the interleukin-1alpha 3′ untranslated region confers risk for hepatocellular carcinoma. Carcinogenesis.

[22] Mennigen JA, Plagnes-Juan E, Figueredo-Silva CA, Seiliez I, Panserat S, Skiba-Cassy S (2014). Acute endocrine and nutritional co-regulation of the hepatic omy-miRNA-122b and the lipogenic gene fas in rainbow trout, Oncorhynchus mykiss. Comp Biochem Physiol B Biochem Mol Biol.

[23] Wang C, Zhao H, Zhao X, Wan J, Wang D, Bi W, Jiang X, Gao Y (2014). Association between an insertion/deletion polymorphism within 3′UTR of SGSM3 and risk of hepatocellular carcinoma. Tumour Biol.

[24] Du Y, Han X, Pu R, Xie J, Zhang Y, Cao G (2014). Association of miRNA-122-binding site polymorphism at the interleukin-1 alpha gene and its interaction with hepatitis B virus mutations with hepatocellular carcinoma risk. Front Med.

[25] Chin LJ, Ratner E, Leng S, Zhai R, Nallur S, Babar I (2008). A SNP in a let-7 microRNA complementary site in the KRAS 3′ untranslated region increases non-small cell lung cancer risk. Cancer Res.

[26] Calin GA, Croce CM (2006). MicroRNA signatures in human cancers. Nat Rev Cancer.

[27] Boesch-Saadatmandi C, Wagner AE, Wolffram S, Rimbach G (2012). Effect of quercetin on inflammatory gene expression in mice liver in vivo - role of redox factor 1, miRNA-122 and miRNA-125b. Pharmacol Res.

[28] Li S, Zhu J, Fu H, Wan J, Hu Z, Liu S (2012). Hepato-specific microRNA-122 facilitates accumulation of newly synthesized miRNA through regulating PRKRA. Nucleic Acids Res.

[29] Mennigen JA, Martyniuk CJ, Seiliez I, Panserat S, Skiba-Cassy S (2014). Metabolic consequences of microRNA-122 inhibition in rainbow trout, Oncorhynchus mykiss. BMC Genomics.

[30] Chang J, Guo JT, Jiang D, Guo H, Taylor JM, Block TM (2008). Liver-specific microRNA miR-122 enhances the replication of hepatitis C virus in nonhepatic cells. J Virol.

[31] Pu Y, Zhang Z, Zhou B, Chen P, Zhang K, Song Y, Gao Q, Wang K, Quan Y, Xi M, Zhang L (2014). Association of an insertion/deletion polymorphism in IL1A 3′-UTR with risk for cervical carcinoma in Chinese Han Women. Hum Immunol.

[32] Zhang Y, Sturgis EM, Sun Y, Sun C, Wei Q, Huang Z, Li G (2015). A functional variant at miRNA-122 binding site in IL-1α 3′ UTR predicts risk and HPV-positive tumors of oropharyngeal cancer. Eur J Cancer.

[33] Guan X, Sturgis EM, Lei D, Liu Z, Dahlstrom KR, Wei Q, Li G (2010). Association of TGF-1 genetic variants with HPV16-positive oropharyngeal cancer. Clin Cancer Res.

[34] Piccirillo JF, Tierney RM, Costas I, Grove L, Spitznagel EL (2004). Prognostic importance of comorbidity in a hospital-based cancer registry. JAMA.

[35] Ji X, Sturgis EM, Zhao C, Etzel CJ, Wei Q, Li G (2009). Association of p73 G4C14-to-A4T14 polymorphism with human papillomavirus type 16 status in squamous cell carcinoma of the head and neck in non-Hispanic whites. Cancer.

